# Piezoelectric polymer gated OFET: Cutting-edge electro-mechanical transducer for organic MEMS-based sensors

**DOI:** 10.1038/srep38672

**Published:** 2016-12-07

**Authors:** Damien Thuau, Mamatimin Abbas, Guillaume Wantz, Lionel Hirsch, Isabelle Dufour, Cédric Ayela

**Affiliations:** 1 Univ. Bordeaux, Laboratoire de l’Intégration du Matériau au Système, UMR 5218, ENSCBP, 16 avenue Pey Berland, 33607, Pessac Cedex, France

## Abstract

The growth of micro electro-mechanical system (MEMS) based sensors on the electronic market is forecast to be invigorated soon by the development of a new branch of MEMS-based sensors made of organic materials. Organic MEMS have the potential to revolutionize sensor products due to their light weight, low-cost and mechanical flexibility. However, their sensitivity and stability in comparison to inorganic MEMS-based sensors have been the major concerns. In the present work, an organic MEMS sensor with a cutting-edge electro-mechanical transducer based on an active organic field effect transistor (OFET) has been demonstrated. Using poly(vinylidenefluoride/trifluoroethylene) (P(VDF-TrFE)) piezoelectric polymer as active gate dielectric in the transistor mounted on a polymeric micro-cantilever, unique electro-mechanical properties were observed. Such an advanced scheme enables highly efficient integrated electro-mechanical transduction for physical and chemical sensing applications. Record relative sensitivity over 600 in the low strain regime (<0.3%) was demonstrated, which represents a key-step for the development of highly sensitive all organic MEMS-based sensors.

Silicon has been the standard material used in microelectronics. However, research in silicon-based micro and nano electro-mechanical systems (MEMS and NEMS) has stalled in the last decade as the field came to maturation^1^. Nevertheless, the broad field of applications of MEMS does not lie exclusively on inorganic materials. Alternative technologies based on tailor made functional materials are currently being explored to satisfy the demand imposed by new applications requiring mechanical flexibility, large area or a certain compatibility with biological systems. Due to their particular properties, organic materials have the potential to replace Si based-MEMS devices, just like organic light emitting diodes (OLEDs) are taking a major share on the world-wide market of smartphone and display industry. MEMS based bio and chemical sensors operating in static mode convert the bio or chemical recognition into a mechanical deflection. This displacement is caused by a mismatch of surface stress between the top and bottom surfaces of the free-standing micro structure as binding of the target analyte occurs. Because of their soft mechanical properties, for a given surface stress, organic MEMS allow a much larger mechanical deflection when compared to inorganic ones. As a result, they exhibit enhanced sensitivity when coupled to an efficient electro-mechanical transduction scheme^2^.

To take advantage of such high sensitivity along with downscaling offered by MEMS-based sensor, hence portability, an integrated electro-mechanical transduction scheme is required. The major electro-mechanical transduction mechanisms can be classified as piezoresistive, capacitive and piezoelectric types. Piezoresistive MEMS offer great mechanical stability. However, they display some drawbacks such as significant power requirement, large temperature dependence offset and nonlinear response^3^. On the other hand, capacitive sensors are relatively easy to fabricate and highly scalable for miniaturization. Both piezoresistive^4^ and capacitive^5^ sensors have been associated with an organic field effect transistor (OFET), demonstrating that organic sensor elements with organic signal processing electronics can be achieved. Similarly, piezoelectric polymers such as polyvinylidene fluoride (PVDF) and its derivative, i.e. copolymer poly(vinylidene fluoride/trifluoroethylene) P(VDF-TrFE), have also been coupled with OFETs for the conception of flexible ferroelectric memory devices^6^,^7^,^8^ or highly sensitive strain sensors^9^. In the latter case, the device combines the efficiency of piezoelectric effect and direct signal amplification. Recently, PVDF-TrFE gated OFET have also been reported as bimodal thin film sensors for simultaneous detection of pressure and temperature, using piezoelectric and pyroelectric effects^10^. Despite the utility of piezoelectric gated OFET devices, research on their use as efficient electro-mechanical transduction scheme in MEMS-based sensors has not been extensively studied. In this respect, Dravid *et al*. reported pioneer work on metal oxide semiconductor field effect transistor (MOSFET) embedded Si cantilever for biological sensing applications^11^,^12^,^13^. Recently, piezotransistive transduction using AlGaN/GaN heterojunctions field effect transistor integrated onto a GaN micro-cantilever has been reported with an ultra-high relative electro-mechanical sensitivity of 8,700. This advanced electro-mechanical transduction allows the detection of nanogram-level explosives with high specificity using a novel surface-based photoacoustic spectroscopy technique^14^. To date, efficient transistor embedded MEMS have been mainly reported for devices based on inorganic materials^11^,^12^,^13^,^14^ since micromachining of advanced polymeric MEMS remains a challenge^15^. However, the development of cutting edge electro-mechanical transduction scheme combining the attractive features of polymers in terms of cost effectiveness, sensitivity and biocompatibility could play an important role in driving new applications. In this regard, Rao’s group was the first to adapt the transistor embedded cantilever configuration into an organic approach^16^. They reported on a standard OFET integrated onto an epoxy resin SU-8 based micro-cantilever. The nonlinear modulation of drain current and mobility observed in their study led to maximum relative variations of drain current and mobility over 250% for 0.1% of applied strain. An extracted strain sensitivity of 10^3^ at low strain level only based on the piezoresistivity of the pentacene organic semiconductor layer was reported^16^. However these results have not been reproduced by subsequent studies investigating the electrical characteristics of OFETs under strain^17^,^18^,^19^,^20^. In another work, Rao *et al*. developed a piezoelectric aluminum doped zinc oxide (Al-doped ZnO) thin film transistor mounted on polymeric cantilever^21^. In that case, a specific low temperature sputtering process of ZnO has been developed, requiring high-cost manufacturing facilities. Although a combination of piezoresistive and piezoelectric effects in enhancing displacement sensitivity was demonstrated, it was characterized by a relatively low sensitivity (116 ppm/nm) and limited to strain sensing applications. Subsequent applications in real time chemical monitoring has never been realized.

In the present work, we report on a novel generation of chemical organic MEMS-based sensors. The successful electro-mechanical transduction was conceived using a piezoelectric polymer gated OFET embedded polyethylene naphthalene (PEN) micro-cantilever and used for steady-state strain and humidity monitoring applications. The presented MEMS-based sensors exhibit a relative electro-mechanical sensitivity of 600. The advantage of the piezoelectric gate dielectric material in improving the electro-mechanical sensitivity by a factor of 18 was clearly verified through electrical characterizations before and after polarization. The devices have been realized from cost effective fabrication processes, eliminating the need for complicated etching steps and lithographic masking required in previously reported studies^21^,^11^,^12^,^13^,^14^,^16^. Also, the high electro mechanical sensitivity obtained has allowed the development of a humidity sensor characterized by a high sensitivity (7500 ppm/%RH) and low limit of detection (0.2%).

## Architecture of the sensor

**Figure 1:**
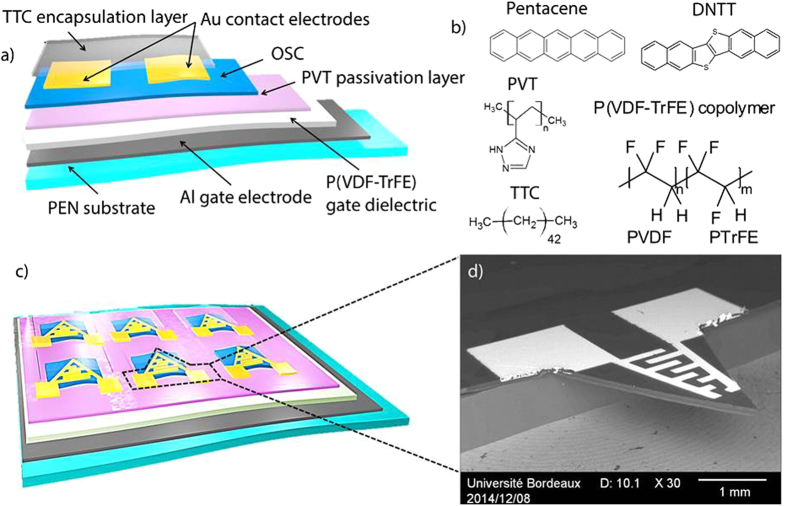
Illustrative schematics, chemical structure and SEM images of the OFET-embedded micro-cantilever MEMS (**a**) Cross-section of the sensor material stacking layers, (**b**) chemical structures of dielectric and semiconductor layers of the OFET, (**c**) schematic of 1.5 cm × 1.5 cm chip containing six OFETs and (**d**) SEM image of a fabricated OFET-embedded cantilever sensor patterned by xurography.

The piezoelectric OFET integrated into a micro-cantilever made of flexible Polyethylene naphthalate (PEN) has a bottom-gate top-contact structure which consists of an Aluminum (Al) gate electrode, P(VDF-TrFE) and poly(1-vinyl-1,2,4-triazole) (PVT) gate dielectric layers, an organic semiconductor (OSC) and a Gold (Au) source-drain (S/D) electrodes. The whole stacking was encapsulated with a thin layer of tetratetracontane (TTC, C_44_H_90_) as schematically illustrated in Fig. 1a. OFET-embedded MEMS were fabricated by combining classical deposition techniques with xurography^22^,^23^,^24^. First, Al was evaporated through shadow mask to pattern gate electrodes. P(VDF-TrFE) piezoelectric copolymer employed as gate dielectric combined with PVT used as dielectric passivation layer were deposited by spin coating^25^. FTIR spectrum of P(VDF-TrFE) thin films was performed in order to ensure a good β phase crystallization of the material. This is illustrated in the Supplementary material (Figure S1). Furthermore, to ensure the piezoelectricity of the gate dielectric material, dynamic characterizations of the piezoelectric gated OFET embedded cantilever showed an efficient resonance behavior by measuring the vibration amplitude of the cantilever using a laser Doppler vibrometer as shown in Figure S2. Afterwards, two well-known p-type OSC (Fig. 1b), pentacene and dinaphtho [2,3-b:2,3-f] thieno [3,2–b] thiophene (DNTT), an air-stable OSC^26^ were thermally evaporated under secondary vacuum with a thickness of 30 nm. 60 nm thick Au contacts were thermally evaporated through shadow masks. The last step consisted in the evaporation of TTC, a long alkane chain molecule generally used as gate dielectric^27^ and employed in this work as a thin encapsulation layer for air stability measurements.

Figure 1c shows the schematic of a 1.5 × 1.5 cm^2^ flexible chip containing six OFETs, and Fig. 1d is a SEM image of the fabricated triangular shaped OFET-embedded micro-cantilevers showing that the device is suspended. One particularly elegant feature is the simplicity of the proposed fabrication process, which eliminates the need for complicated etching steps and lithographic masking required in silicon processing. In addition, a major advantage of such processing approaches is the reduction in cost to fabricate MEMS. The presented fabrication process provided a 95% yield of operating devices as shown by Figure S3.

## Electro-mechanical transducer

As deposited, P(VDF-TrFE) gate dielectric layer does not possess a high remnant polarization (*P*_*r*_) and behaves like a normal gate dielectric material. In order to induce piezoelectricity, a polarization process is required so that polarization states can be generated inside the material. This can be accomplished by the application of an electric field. As indicated in the electrical characterization responses shown in Figure S4, this leads to a hysteresis loop in the polarization versus electric field, (P-E). In our case, a poling electric field of 100 MV.m^−1^ has been applied between the gate electrode and short-circuited S/D electrodes. Voltage bias has been applied for 5 minutes. It is noted that the electric field is not homogeneous in the dielectric layer due to the channel. As a result, polarization characterizations on planar capacitors (metal/insulator/metal) have also been performed to determine the optimal *P*_*r*_ achievable with this dielectric. *P*_*r*_ for capacitor and transistor type devices was measured to be 8.2 and 2.4 μC.cm^−2^, respectively. The reduction of *P*_*r*_ and the tilt of the P-E curve in transistor configuration were attributed to channel geometry. In fact, the OSC layer which acts as the top electrode during polarization is not conductive enough to ensure an equipotential. However, electrical polarization of our fabricated devices exhibits excellent time stability^28^.

**Figure 2:**
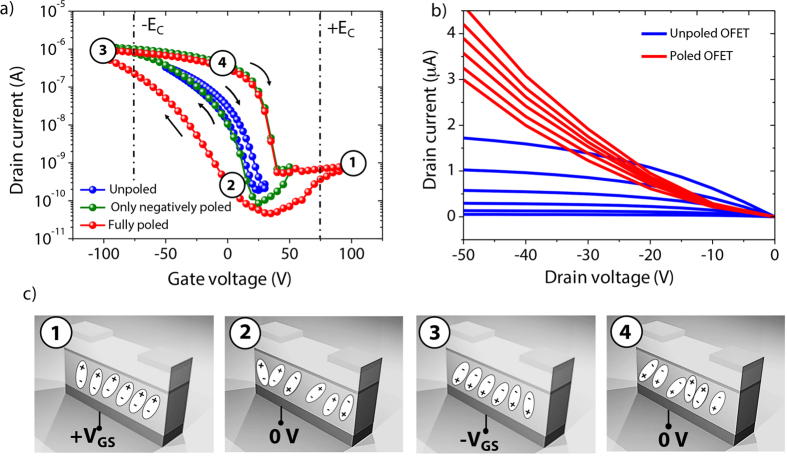
Electrical characterization and piezoelectricity (**a**) Transfer characteristics of piezoelectric OFET based on pentacene under various *V*_*GS*_ sweep windows (at *V*_*DS*_ = −5 V) showing that poling of the piezoelectric gate dielectric is achievable under sweeps at high voltages. Black arrows represent the scanning directions. Round circles 1, 2, 3 and 4 correspond to specific electrical states of the P(VDF-TrFE), (**b**) corresponding output characteristics for different *V*_*GS*_ from 0 to −50 V by steps of −10 V and (**c**) four specific electrical states of the OFET at different *V*_*GS*_ voltages corresponding to: state 1: positive polarization, the transistor is off; state 2: no polarization, transistor is still off due to remnant polarization preventing hole injection; state 3 negative polarization, the transistor is on; and state 4 no polarization, transistor is still on due to remnant polarization inducing channel hole accumulation.

Figure 2a and b compare respectively the transfer and corresponding output characteristics of a fabricated OFET-embedded cantilever for different *V*_*GS*_ sweeps. Note that, except where otherwise stated, measurements were performed in air at room temperature and at 50% of relative humidity on OFETs made of P(VDF-TrFE) as gate dielectric and pentacene as semiconducting layer. The fabricated devices exhibited excellent ambient air stability, and all electrical measurements presented in this work were performed under ambient conditions. The current on/off ratio exceeded 10^4^ and the estimated linear-regime mobility was 0.1 cm^2^.V^−1^.s^−1^ at *V*_*GS*_ = −50 V. A large dielectric thickness (2 μm thick of P(VDF-TrFE) + 50 nm thick layer of PVT) and related low capacitance (~2.5 nF.cm^−2^) measured with an Agilent E5061B network analyzer led to high operating voltages. Obviously, such relatively high operating voltage can be remarkably decreased by reducing the thickness of the gate dielectric layer^29^,^30^. The primary information obtained from Fig. 2a is the increase in *I*_*DS*_ hysteresis by increasing the *V*_*GS*_ sweep window. The hysteresis window of the transfer characteristics (at *V*_*DS*_ = −5 V) is due to *P*_*r*_ induced in the piezoelectric gate dielectric layer by the electric field generated during *V*_*GS*_ sweep measurement. The arrangement of the H-F dipoles in the piezoelectric material as a function of applied *V*_*GS*_ is illustrated in Fig. 2c. A more detailed explanation of the polarization mechanism which takes place in the PVDF-TrFE gate dielectric layer can be found in supporting material S5. In addition, we assumed that this phenomenon was also the cause for the linearization of the output curves. The *I*_*DS*_ value of the polarized device (in the state 4 region) doubles from almost 2 μA to over 4 μA as shown in Fig. 2b.

**Figure 3:**
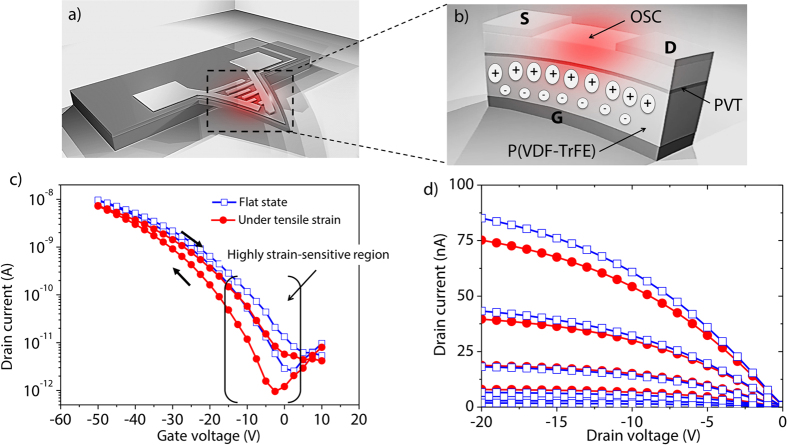
Schematics and electrical characteristics of the sensor under strain (**a**) Schematic of the cantilever MEMS bent due to applied tensile strain with the red region highlighting the surface strain experienced by the piezoelectric OFET mounted onto the cantilever device, (**b**) cross sectional view of the piezoelectric OFET based transducer under tensile strain changing the distance between hydrogen and fluorine atoms in the P(VDF-TrFE) layer which leads to a depletion of positive charge in the p-type semiconductor and (**c**) transfer characteristics (at *V*_*DS*_ = −5 V) of a polarized OFET-embedded cantilever in a flat state and under 0.28% of tensile strain and (**d**) corresponding output characteristics for different *V*_*GS*_ from −2 V to −20 V by steps of −2 V.

In the first set of experiments, the performances of the electro-mechanical transduction scheme were evaluated by testing the devices as strain sensors. To do so, mechanical loads were applied at the tip of the triangular MEMS using micromanipulators miBot from Imina Technology SA. Under applied mechanical loads (Fig. 3a), the OFET-embedded cantilever bends and consequently results in a polarization of the gate induced by the piezoelectric layer (Fig. 3b). Then, the piezoelectric effect causes a change of charge density in the semiconductor channel of the OFET, leading to amplified modulation of *I*_*DS*_. In other words, the sensing mechanism of the electro-mechanical transducer originates from the piezoelectric material itself, which affects the electrical behavior of the transistor as signature of a mechanical event.

Concretely, the effects of strain on the transfer and output characteristics of polarized OFET-embedded cantilevers were investigated. The strain generated on the surface of the OFET-embedded cantilevers was determined by measuring the deflection of the cantilever using an optical profilometer (Veeco NT9080). Subsequently, the respective *I*_*DS*_ measurements were recorded simultaneously using a Keithley 4200 semiconductor analyzer to determine electro mechanical sensitivities. Figure 3c is a plot of the transfer characteristics of positively polarized piezoelectric OFET-embedded MEMS at rest and under a tensile strain of 0.28%. Figure 3d depicts the corresponding output characteristics. From Fig. 3c, one important observation is the significant shift of *V*_*th*_ towards the negative values as a function of applied strain, estimated at −2.5 V and −7.5 V at 0 and 0.28% of tensile strain, respectively. This can be explained by the direct piezoelectric effect that induces a change in surface charge density at the interface with the organic semiconductor in response to an external mechanical strain. We stress that, at higher gate voltage, the charge density generated by the piezoelectric gate dielectric remains low compared to charges induced by the gate voltage. Consequently, modulation of the OFET’s behavior by the piezoelectric effect is clearly prominent at *V*_*GS*_ close to *V*_*th*_. One should note that both transfer and output characteristics completely came back to the initial state after the tensile strain was set back to zero, thereby demonstrating the good reversibility of the sensor as shown by Figure S6. Also, the slight subthreshold slope change between the two curves was assigned to a change of capacitance and interface trap densities.

## Effects of Electrical polarization

**Figure 4:**
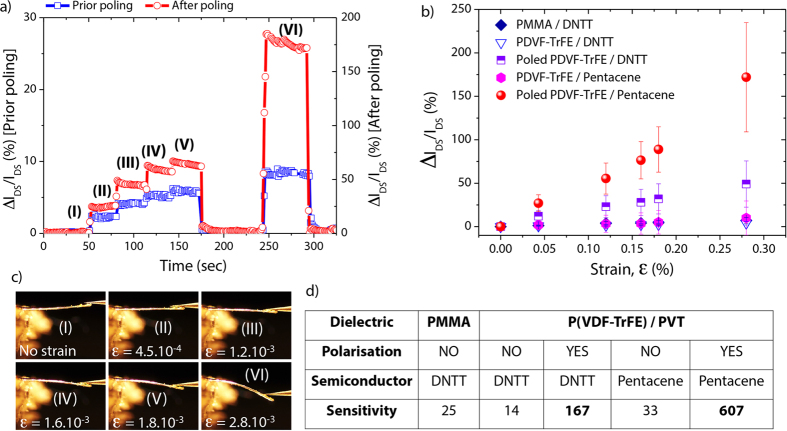
Real-time monitoring of strain and electro-mechanical performances (**a**) Real time measurements of relative changes of drain current (Δ*I*_*DS*_*/I*_*DS*_) in a device submitted to different applied tensile strain of 0, 0.045, 0.12, 0.16, 0.18 and 0.28% (**b**) relative change of drain current as a function of applied strain ε for different coupled gate dielectric/OSC layers in polarized OFET-embedded cantilever, (**c**) optical images of the cantilever profile under different tensile strain levels corresponding to the state I to VI and (**d**) comparison of strain sensitivity [(Δ*I*_*DS*_*/I*_*DS*_)*/*Δε] for different stacking of gate dielectric and OSC materials.

To further investigate the influence of electrical polarization on the electro-mechanical responses of the fabricated OFET-embedded cantilevers, we ran tensile strain cycle measurements. Concretely, crescent forces were applied and released to both pristine and polarized devices while recording simultaneously drain current variations (V_DS_ = −5 V and V_GS_ = −50 V) (Fig. 4a). As shown in Fig. 4a, steady state relative drain current variations (Δ*I*_*DS*_*/I*_*DS*_) of pristine devices were small compared to those of polarized ones namely, 9% and 170%, respectively for an identical applied strain value of 0.28%. These values correspond to the average values of drain current recorded over 60 seconds taking into account the small drift of current due to resistive losses in the dielectric layer, combined to the viscoelastic response of the materials. Their corresponding electro-mechanical sensitivities defined as ((Δ*I*_*DS*_*/I*_*DS*_)/ε) were calculated to be 33 and over 600. The large enhancement of the strain sensitivity close to the *V*_*th*_ by a factor 18 after poling clearly demonstrated the benefit offered by the piezoelectric effect. Indeed, change of polarization of P(VDF-TrFE) affects the charge density in the pentacene layer. Figure 4b plots the average Δ*I*_*DS*_*/I*_*DS*_ responses and their corresponding standard deviations as a function of tensile strain for five devices of each material stacking configurations listed in Fig. 4d. Two small molecules, pentacene and DNTT, were tested as OSC, along with two dielectric materials, PMMA used as control passive dielectric and piezoelectric copolymer P(VDF-TrFE). Transfer and output characteristics of OFETs made from each material stacking are illustrated in Figure S7. All measurements presented quasi-linear *I*_*DS*_ changes as a function of the applied surface strain. As already mentioned, intrinsically, P(VDF-TrFE) is not piezoelectric and therefore behaves like a passive gate dielectric such as PMMA. This behavior was confirmed by the homogeneity of the sensitivity of OFETs made of PMMA/DNTT, unpoled P(VDF-TrFE)/DNTT and unpoled P(VDF-TrFE)/pentacene determined to be 25, 14 and 33, respectively, as summarized in Fig. 4d. Nevertheless, once the P(VDF-TrFE) layer was polarized, one can observe a significant improvement of the strain sensitivity by a factor 18 regardless of the OSC materials. Among polarized devices, pentacene based OFETs presented the largest sensitivities. It is well-known that strain-sensitivity of OSC is highly dependent on its morphological structure^18^,^19^. Thus, the record sensitivities observed in our piezoelectric OFET-embedded MEMS was due to a combination of piezoelectricity induced in the active gate dielectric and a strain dependent mobility of the OSC. In parallel, we investigated the influence of strain on the P(VDF-TrFE) dielectric capacitance. Sandwiched capacitor structures of similar active area have been fabricated and characterized under identical tensile strain. All tested devices showed no more than 2% of relative capacitance variations (Δ*C/C*) as shown by Figure S8 for similar strain (0.28%). Such small variations were clearly negligible considering the large *I*_*DS*_ changes obtained for polarized devices (from 45% to 170%).

To put the benefits of the proposed piezoelectric gated OFET transduction in perspective, we compared the obtained sensitivity with different piezoresitive/piezotransitive schemes. Electro-mechanical performances of our sensors (600) are superior to those of classical Si piezoresistances^31^ or piezorestive CNT based nanocomposites^3^ reported to be around 200. They are also higher than those of graphene piezoresistive thin films (<300)^32^. Although higher sensitivities have been reported for nanowire-based piezoresistive devices such as CNT nanowires (>1,000)^31^, the ease of fabrication of our sensor which can also operate in the low frequency range of few kHz, as shown in Figure S2, should have a significant impact on a novel generation of highly sensitive transduction in a variety of sensing applications. Secondly, and perhaps more importantly is the contribution of the piezoelectric gate dielectric layer on the sensors’ performances. While the intrinsic piezotransistive sensitivities of pentacene and DNTT based OFETs presented here are in good agreement with recently reported values (10–50)^17^,^18^,^19^,^20^, the polarization of the piezoelectric gate dielectric offers a large enhancement of the sensor’s sensitivity. As a result, the technological demand for highly sensitive, low cost sensing devices in some applications can be addressed.

## Example of application: humidity sensing

**Figure 5:**
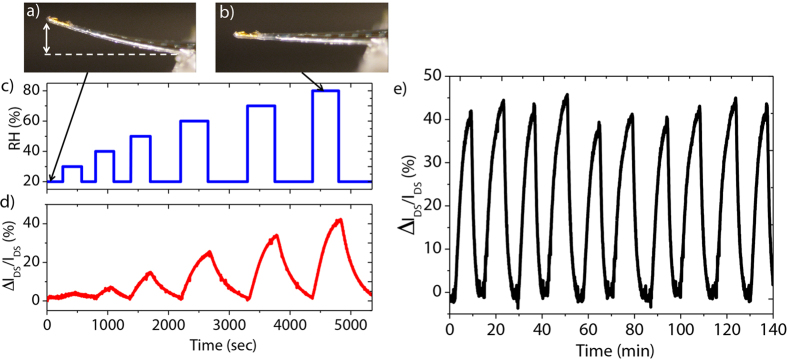
Real-time monitoring of humidity (**a**) Optical profile of the OFET-embedded sensor that has been functionalized with a hydrogel layer on top at 20% of relative humidity, (**b**) at 80% of relative humidity, (**c**) relative humidity control as a function of time, (**d**) relative changes of drain current (Δ*I*_*DS*_*/I*_*DS*_) plotted versus elapsed time for different levels of relative humidity and (**e**) (Δ*I*_*DS*_*/I*_*DS*_) plotted versus elapsed time for rectangular cycles of 20% and 80% of relative humidity.

As a simple model of sensing application, the fabricated MEMS devices were tested as humidity sensors. Herein, the devices were coated with a hydrogel thin film employed as the sensitive layer. The hydrogel was synthesized by free radical polymerization of hydroxyethyl-methacrylate (HEMA) and ethylene-glycol-dimethacrylate (EGDMA) monomers where EGDMA acts as a cross-linker. It has the ability to swell in the presence of humidity. In addition to hydrogels offering improvements in sensitivity, selectivity and response time, their compatibility with printable technology at extremely low cost makes them an attractive material for sensing applications^33^. Often used as sensing layer in optical-based sensing devices^34^,^35^, within this work, we integrated them in electrically transduced sensors. The large volume change due to water molecule absorption leads to surface strain experienced by the MEMS-based sensor due to bi-layer effect (Fig. 5a and b) that, in turn results in large *I*_*DS*_ modulations. As depicted in Fig. 5c,d, increasing RH significantly increases linearly the relative changes of drain current (Δ*I*_*DS*_ */I*_*DS*_). Herein, an elapsed time of 500 seconds has been chosen for each humidity level steps due to the observation of a plateau in the sensors’ response afterwards (Figure S9). The sensitivity of our sensor has been measured to be 7500 ppm/%RH with an extracted limit of detection of 0.2%RH. Such sensitivity is one of the largest value reported in the literature on MEMS-based humidity sensors^36^,^37^. The good repeatability and reversibility of the sensing properties are illustrated by the homogeneous relative drain current variations in the order of 42% over ten cycles of RH variations (Fig. 5e). As expected, the sensors’ response under increasing and decreasing RH level showed good overlapping of forward and backward curves proving the absence of hysteresis in the sensor response (Figure S10). An increase of humidity from 20% to 80%, was found to correspond to 0.1% tensile strain experienced by the cantilever from optical image analysis of the cantilever deflection. As an example, a video of the sensor measuring RH in real time can be seen in Supplementary Video 11. The relative change of drain current shows excellent agreement with previous values obtained in the case of strain sensing where an average value of (Δ*I*_*DS*_*/I*_*DS*_) of 47% was estimated for such material stacking and such applied tensile strain level (see Fig. 4b). To verify the influence of humidity on the intrinsic performance of OFETs, an encapsulated pentacene based OFET without the hydrogel coating has shown relative drain current variations of only 4% (Figure S12). These results highlight the benefits offered by the MEMS configuration where mechanical deflection amplifies the sensor’s response. This result confirmed the ability of the piezoelectric OFET-embedded MEMS to monitor steady state sensing events and thus, show promise for future monitoring of complex sensing events, such as biological analysis.

## Conclusion

In the present work, the realization of a piezoelectric polymer P(VDF-TrFE) gated OFET as effective electro-mechanical transduction scheme in organic micro-cantilever for physical and chemical sensing applications has been reported. The benefits of incorporating an active piezoelectric gate dielectric material in an OFET have been demonstrated in enhancing MEMS sensors’ strain sensitivity by a factor of 18. The presented sensors exhibit a linear relative strain sensitivity ((Δ*I*_*DS*_*/I*_*DS*_)/Δε) of 600 at low strain level (below 0.3%). In the case of humidity monitoring, the sensor exhibited also excellent performance with a measured sensitivity of 7500 ppm/%RH and an extracted limit of detection of 0.2%RH. The developed electro-mechanical transduction scheme herein presented should guide the design of a large number of low-cost MEMS-based sensors targeting high sensitivity for chemical and biological sensing applications.

### Experimental Section

*PVDF-TrFE:* The 70:30 mol% copolymer P(VDF-TrFE), purchased from Piezotech ARKEMA in particles form, was dissolved under stirring at room temperature for at least 2 h in 2-butanone. The solution was spin coated at a rotation speed of 5000 rpm and subsequently annealed at 140 °C for 2 h in order to evaporate the solvent and to increase crystallinity. The crystallinity of the PVDF-TrFE has been calculated to be 52.7%. It has been extracted from the area of the melting point and Curie temperature of the DSC measurement. The thickness of the polymer films was controlled for the specific solvent by two parameters: the concentration of the polymer dissolved in the solvent and the rotation speed during the deposition.

#### Fabrication of the piezoelectric based OFET-embedded MEMS

OFETs were fabricated on flexible 50 μm thick Polyethylene naphthalate (PEN) substrates via a simple, low-cost and consistent fabrication process. To reduce their surface roughness, PEN substrates were cleaned in acetone, isopropanol and deionised water bath successively for 5 minutes and then cured at a temperature of 180 °C for 2 h to allow the formation of oligomer at the film surface. The surface roughness of the PEN substrates after thermal treatment was reduced to 2.72 nm rms. Then, 80 nm thick Aluminium (Al) gate electrodes were deposited by electron beam evaporation at low pressure (1 × 10^−7^ mbar) on the substrates through shadow mask. 15 wt% of P(VDF-TrFE) piezoelectric copolymer dispersed in 2-butanone was spin coated and subsequently thermally cured at 140 °C for 2 h to obtain a 2 μm thick gate dielectric layer. Afterwards, a plassivation layer of 50 nm thick of poly(1-vinyl-1,2,4-triazole) (PVT) layer was additionally deposited to smoothen the P(VDF-TrFE) surface roughness and to decrease interface trap densities. Subsequently, pentacene or dinaphtho [2,3-b:2,3-f] thieno [3,2–b] thiophene (DNTT), two standard p-type OSC materials were evaporated at a base pressure of 1.10^−6^ mbar; the film thickness and the deposition rate (0.6 nm.min^−1^) were monitored by a quartz crystal microbalance placed next to the sample. The materials were chosen owing to their relatively high hole mobility. Gold (Au) (Source/Drain) contacts were patterned by thermal evaporation through shadow mask. An encapsulation layer of 150 nm thick tetratetracontane (TTC, C_44_H_90_), a long chain alkane molecule was evaporated through shadow mask. Finally, the micro-cantilevers were patterned by xurography using a vinyl cutting machine (Graphtec Craft ROBO Pro CE 5000–40).

#### T-F analyser characterization

Polarization hysteresis loops were measured by applying a triangular voltage waveform with a frequency of 100 Hz between the bottom and the grounded top electrodes. The data has been plotted with P-E curve standard graph as shown in Figure S4.

#### Hydrogel formulation

The hydrogel was synthesized by free radical polymerization of hydroxyethyl-methacrylate (HEMA) and ethylene-glycol-dimethacrylate (EGDMA) monomers where EGDMA acts as a cross-linker. HEMA and EGDMA were both purchased from Sigma Aldrich. The polymerization process was initiated by ultraviolet light. Note that we use 2-Hydroxy-4-(2-hydroxyethoxy)-2-methylpropiophenone as photo initiator. The polymerization process was performed in a glove box under inert atmosphere to avoid undesirable interaction of oxygen during polymerization by reacting or forming complexes with the photo initiator.

## Additional Information

**How to cite this article**: Thuau, D. *et al*. Piezoelectric polymer gated OFET: Cutting-edge electro-mechanical transducer for organic MEMS-based sensors. *Sci. Rep.* **6**, 38672; doi: 10.1038/srep38672 (2016).

**Publisher's note:** Springer Nature remains neutral with regard to jurisdictional claims in published maps and institutional affiliations.

## Supplementary Material

Supplementary Video 10

Supplementary Information
