# Supplementary material for: Piezoelectric polymer gated OFET: Cutting-edge electro-mechanical transducer for organic MEMS-based sensors
Source: Sci Rep. 2016 Dec 7;6:38672. doi: 10.1038/srep38672 (PMC5141423; doi:10.1038/srep38672)
Supplement: Supplementary Information [file srep38672-s2.doc]

Supplementary Information

Piezoelectric polymer gated OFET: Cutting-edge electro-mechanical transducer for organic MEMS-based sensors

Damien Thuau, Mamatimin Abbas, Guillaume Wantz, Lionel Hirsch, Isabelle Dufour and Cédric Ayela


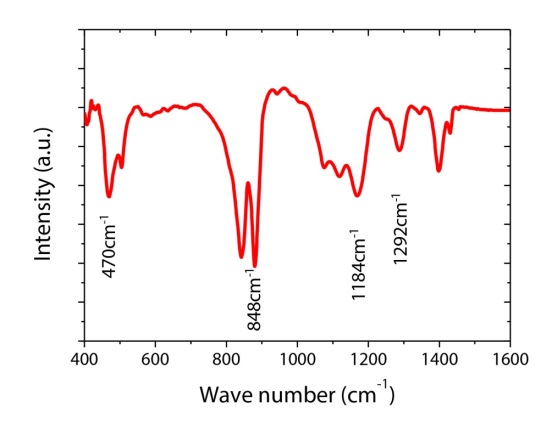


Figure S1. FTIR spectrum of P(VDF-TrFE) layer

Four significant intense bands at 470 cm^−1^, 848 cm^−1^, 1184 cm^−1^ and 1292 cm^−1^ representative of the *β* phase of P(VDF-TrFE) were obtained. The 470 cm^−1^ and 1292 cm^−1^ bands were assigned to the CF_2_ bending mode within TTT segments of the chain and the wagging vibration of CH_2_, respectively; whilst the bands at 848 cm^−1^ and 1184 cm^−1^ were assigned to CF_2_ symmetric stretching mode.

Figure S2. Magnitude of displacement and phase of the resonant spectrum of the piezoelectric polymer P(VDF-TrFE) gated OFET embedded polymeric MEMS micro-cantilever resonator

Figure S3. Transfer curves of 19 piezoelectric based OFET mounted on organic micro-cantilever for *V_DS_* =-5V

Figure S4. (P-E) hysteresis loop illustrating the polarization induced in the P(VDF-TrFE) layer as a function of applied electric field measured with a T-F analyzer

Electrical behavior of ferroelectric / piezoelectric P(VDF-TrFE) OFETs can be switched from one state to another by applying *V_GS_* values corresponding to electric fields larger than the coercive field of P(VDF-TrFE). This explains the hysteresis asymmetry observed in the transfer characteristics of the negatively polarized OFET. In this case, *V_GS_* was swept from +50 V to -100 V, exceeding the voltage corresponding to the negative coercive field but not the positive one. Obviously, this enlargement of the transfer curve is caused by an increase of threshold voltage (*V_th_*) of positively polarized device. *V_th_* shift can be explained by the voltage required to compensate for depolarizing before charge injection. The arrangement of the H-F dipoles in the piezoelectric material as a function of applied *V_GS_* illustrated in Figure 2c can be described as follow: Initially, at positive *V_GS_*, the transistor is off (state 1) since the hydrogen atoms are pointing towards the OSC layer, hence no charge injection. Then, sweeping *V_GS_* from positive to negative values turns-on the transistor. A rapid increase in *I_DS_* is observed due to excess holes in the OSC following hole injection (from state 2 to state 3). When *V_GS_* returns to zero, *I_DS_* still remains high (state 4) due to remnant polarization. The subsequent application of positive *V_GS_* to the device gradually switched the dipole moments, decreasing effective field for charge injection (state 1).

Supporting information S5. Principle mechanism of the polarization of the PVDF-TrFE based OFET

Figure S6. Transfer characteristics (at *V_DS_* = -5 V) of a positively polarized OFET-embedded cantilever for three tensile strain cycles between 0 and 0.28 %

Figure S7. Electrical characteristics of OFET: a) transfer curve of DNTT/PMMA based OFET at V_DS_=-2V, b) corresponding output curves for different gate voltage, c) transfer curves of pentacene/PVDF based OFET before and after electrical polarization, d) corresponding output curves for V_GS_ from 0V to -50V by steps of -10V, e) transfer curves of DNTT/PVDF based OFET before and after electrical polarization, d) corresponding output curves for V_GS_ from 0V to -50V by steps of -10V

Figure S8. a) Capacitance as a function of frequency for different tensile strain applied to the OFET-embedded cantilever-based MEMS, b) Relative variation of capacitance (Δ*C/C*) as a function of strain and c) capacitance variations for three tensile strain cycles between 0 and 0.28%

Figure S9. Response time of the humidity sensor for an increase of RH from 20 to 80%

Figure S10. Relative drain current variation under increasing and decreasing RH level showing the absence of hystersis in the sensor response

Video 11. Steady-state relative humidity sensing


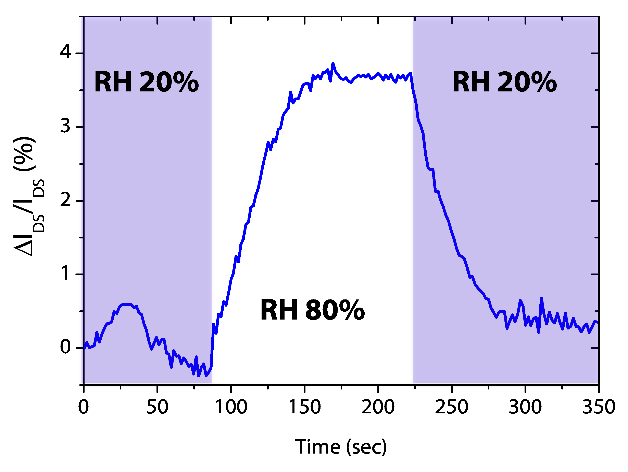


Figure S12. Relative drain current modulations for pentacene based OFET without the hydrogel coating
